# EZ Entropy: a software application for the entropy analysis of physiological time-series

**DOI:** 10.1186/s12938-019-0650-5

**Published:** 2019-03-20

**Authors:** Peng Li

**Affiliations:** School of Control Science and Engineering, Shandong University, 17923 Jingshi Road, Jinan, 250061 Shandong People’s Republic of China

**Keywords:** Entropy, Software, Program, MATLAB

## Abstract

**Background:**

Entropy analysis has been attracting increasing attentions in the recent two or three decades. It assesses complexity, or irregularity, of time-series which is extraordinarily relevant to physiology and diseases as demonstrated by tremendous studies. However, the complexity can hardly be appreciated by traditional methods including time-, frequency-domain analysis, and time-frequency analysis that are the common built-in options in commercialized measurement and statistical software. To facilitate the entropy analysis of physiological time-series, a new software application, namely *EZ Entropy*, was developed and introduced in this article.

**Results:**

*EZ Entropy* was developed in MATLAB^®^ environment. It was programmed in an object-oriented style and was constructed with a graphical user interface. *EZ Entropy* is easy to operate through its compact graphical interface, thus allowing researchers without knowledge of programming like clinicians and physiologists to perform such kind of analysis. Besides, it offers various settings to meet different analysis needs including (1) processing single data recording, (2) batch processing multiple data files, (3) sliding window calculations, (4) recall, (5) displaying intermediate data and final results, (6) adjusting input parameters, and (7) exporting calculation results after the run or in real-time during the analysis. The analysis results could be exported, either manually or automatically, to comma-separated ASCII files, thus being compatible to and easily imported into the common statistical analysis software. Code-wise, *EZ Entropy* is object-oriented, thus being quite easy to maintain and extend.

**Conclusions:**

*EZ Entropy* is a user-friendly software application to perform the entropy analysis of time-series, as well as to simplify and to speed up this useful analysis.

**Electronic supplementary material:**

The online version of this article (10.1186/s12938-019-0650-5) contains supplementary material, which is available to authorized users.

## Background

It is a contemporary challenge to identify characteristics from physiological signals or time-series that are relevant to aging or disease progression. Efforts have been made in using moments, either lower-ordered or higher-ordered, frequency-domain analysis and time-frequency analysis to mine the data. But results based upon these traditional approaches so far are not quite satisfactory. One of the possible reasons might be that those that could be captured by these traditional analyses are usually also visually identifiable which is not the case for physiological data. What is relevant to physiology or disease may be hiding deep behind the fluctuations of the signal. In the most recent two or three decades, researchers from interdisciplinary fields have proposed the concept of nonlinear dynamical analysis, and from then on lines of evidence have demonstrated the unique power of various nonlinear dynamical characteristics in this regard [1–4].

Amongst the vast number of different nonlinear dynamical methods, entropy analysis has gained broad attention, and, thereby, has witnessed its wide suitability in time-series of limited length, short length, or even extremely short length [5–17]. However, lack of knowledge in programming has been hindering most clinicians, and physiologists etc., from performing such kind of analysis. Although many researchers have published or shared open-source codes either in formal publications [14] or in various online repositories [18–24], neither of them is really user-friendly as it still requires users to have at least some basic coding training in order to apply these codes.

The aim of this current work was to introduce a graphical interface-based software application, namely *EZ Entropy*, which was dedicatedly developed for the purposes of calculating the entropy of physiological time-series (and certainly other types of physical time-series). *EZ Entropy* is quite easy to operate with only few clicks to do the calculations of several common entropy measures. And it comes with a couple of different calculation options so as to adjust input parameters, to batch process multiple signal recordings, to do the calculations through sliding windows, to display intermediate results, and so on. In the section below, I will start with a short review of different entropy measures and in “Implementation” section, this software will be described in details.

## Entropy measures: a brief historical review

Entropy came from a field called thermodynamics, a branch of physics. It was initially proposed as a state function of a thermodynamic system which depends only on the current state while is independent of how the state has been achieved. Later on, this macroscopic concept was found to be meaning uncertainty or disorder microscopically measuring the possible number of microscopic states in which the system could be arranged.

Being directly analogous to the microscopic thermodynamic definition, information entropy was defined to quantify the average information content to be expected from an event, or, in other words, the uncertainty or unpredictability of the state of an event. In time-series analysis field, this concept had triggered the idea of assessing the unpredictability of the evolution of dynamical systems, specifically the Kolmogorov entropy of time-series (or Kolmogorov–Sinai entropy, a specific case of Kolmogorov entropy with the time delay factor being equal to unity) [25].

The calculation of Kolmogorov–Sinai entropy was highly noise-sensitive and needed to solve limits, making it infeasible for real world applications. In 1991, Pincus proposed an approximation algorithm—approximate entropy (ApEn) [5] which showed reasonable robustness against noise and was relatively stable for medium length time-series [6, 26]. Since then, ApEn has been successfully applied to many physiological data and has helped gain a lot of valuable, additional insights into physiological controls [27–32]. It has also been introduced to mechanical and many other physical systems [33, 34]. In the meantime, investigators have also been aware of its deficiencies in terms of strong dependence on input parameters and unreliable performance in short-length data, and have thereby proposed a couple of solutions to improve its performance. In the subsections below, I will briefly summarize the algorithms of ApEn and two common refined ApEn metrics, namely sample entropy (SampEn) and fuzzy entropy (FuzzyEn). Besides, I will also introduce several other entropy-like metrics that were proposed in similar contexts.

### ApEn and refined ApEn algorithms

Entropy metrics, in general, quantify the similarity of motifs (which are called vectors in the state space representation) as a proxy to the unpredictability or irregularity of a time-series. For a time-series of *N* points $\mathbf u ={u(i), 1 \le i \le N}$, its *m*-dimension state space representation is

1 $$\begin{aligned} \mathbf X _{m}(i) = \{u(i), u(i+\tau ), \ldots , u(i+(m-1)\tau )\}, \end{aligned}$$

where $1 \le i \le N-m\tau$ and $\tau$ is the time delay parameter, which, together with the dimension parameter *m*, determine how well the state space reconstruction of the dynamical system is. In order to quantify whether two vectors, namely, $\mathbf X _{m}(i)$ and $\mathbf X _{m}(j)$, are similar, the Chebyshev distance between the two vectors is calculated as follows:

2 $$\begin{aligned} {d[\mathbf X _{m}(i), \mathbf X _{m}(j)] = \max _{0 \le k \le m-1}(|u(i+k) - u(j+k)|)}. \end{aligned}$$

The difference between ApEn and its refined algorithms lies basically in the means that is used to assess the overall similarity of each pair of vectors in the state space, which, in turn, leads to different performance.

#### ApEn

In ApEn, the percentage of the vectors $\mathbf X _{m}(j)$ that are within *r* of $\mathbf X _{m}(i)$ is calculated by $C_{i}^{(m)}(r) = \frac{N_i^{(m)}(r)}{N-m\tau }$, where $N_i^{(m)}(r)$ indicates the number of *j*’s that meet $d_{i,j} \le r$, and $1 \le j \le N-m\tau$. And then the average of the percentage over $1 \le i \le N-m\tau$ after logarithmic transform is defined by $\Phi ^{(m)}(r)=\frac{1}{N-m\tau }\sum _{i=1}^{N-m\tau }\ln C_i^{(m)}(r)$. In a similar way, $\Phi ^{(m+1)}(r)$ is defined after increasing the dimension to $m+1$. Then, the ApEn value of the time-series $\mathbf u$ can be calculated by [5]:

3 $$\begin{aligned} \text {ApEn}(m,\tau ,r) = \Phi ^{(m)}(r) - \Phi ^{(m+1)}(r). \end{aligned}$$

#### SampEn

In SampEn, self-matches are excluded when calculating the percentage of the vectors $\mathbf X _{m}(j)$ that are within *r* of $\mathbf X _{m}(i)$, i.e., $A_{i}^{(m)}(r) = \frac{N_i^{(m)}(r)}{N-m\tau -1}$, where $N_i^{(m)}(r)$ indicates the number of *j*’s that meet $d_{i,j} \le r$, and $1 \le j \le N-m\tau , j\not =i$. The average of the percentage $A_{i}^{(m)}(r)$ over $1 \le i \le N-m\tau$ is then defined by $\Psi ^{(m)}(r)=\frac{1}{N-m\tau }\sum _{i=1}^{N-m\tau }A_i^{(m)}(r)$. In a similar way, $\Psi ^{(m+1)}(r)$ is defined after increasing the dimension to $m+1$. The SampEn value of the time-series $\mathbf u$ can be calculated by [8]:

4 $$\begin{aligned} \text {SampEn}(m,\tau ,r) = -\ln \frac{\Psi ^{(m+1)}(r)}{\Psi ^{(m)}(r)}. \end{aligned}$$

#### FuzzyEn

FuzzyEn is methodologically the same to SampEn except that it replaces the percentage of vectors $\mathbf X _m(j)$ that are within *r* of $\mathbf X _m(i)$ with the average degree of membership which offers reliability especially for short-length data. Specifically, for a given fuzzy membership function $e^{-\ln (2)(x/y)^2}$, $A_i^{(m)}=\frac{\sum _{j=1,j\not =i}^{N-m\tau }e^{-\ln (2)(d_{i,j}/r)^2}}{N-m\tau -1}$ is applied in FuzzyEn [11].

### Other entropy-like metrics

#### Conditional entropy (CE)

CE evaluates the information carried by a new sampling point given the previous samples by estimating the Shannon entropy of the vectors with length *m* and vectors with new sampling point added (i.e., with length $m+1$) [7]. CE first coarse-grains the time-series $\mathbf u ={u(i), 1 \le i \le N}$ with a quantification level of $\xi$ (i.e., coarse-grain resolution is $\frac{\max (u)-\min (u)}{\xi }$). Instead of using the original time-series, it reconstructs the coarse-grained time-series into the state space. After the reconstruction, the signal motifs of length *m* and these of length $m+1$ are codified in decimal format (i.e., the first element in the motif of length *m* has a weight of $\xi ^{(m-1)}$ and so on), rendering the sequence of motifs series of integer numbers. The frequency of each possible integer value can then be calculated. CE is defined by the difference between the Shannon entropy of the motif of length $m+1$ and that of the motif of length *m*. In [16], Shi et al. have a beautiful summary of the details of CE algorithm with mathematical formulas.

#### Permutation entropy (PermEn)

PermEn assesses the diversity of ordinal patterns within a time-series. For each signal motif of length *m*, it defines a permutation vector ${\varvec{\pi }}$ by indexing its elements in an ascending order. Then, the frequency of each permutation pattern $\pi _{j}\,(1 \le j \le m!)$ can be calculated. The PermEn of the original time-series is defined by the Shannon entropy of permutation patterns [10, 16].

#### Distribution entropy (DistEn)

Instead of only calculating the probability of similar vectors (i.e., the percentage of the vectors $\mathbf X _{m}(j)$ that are within *r* of $\mathbf X _{m}(i)$), DistEn takes full advantage of the matrix $d_{i,j},1 \le i,j \le N-m\tau$ defined in SampEn algorithm by estimating the Shannon entropy of all distances. Specifically, the empirical probability density function of the distance matrix $d_{i,j}$ except the main diagonal, i.e., $i\not =j$, is first estimated by histogram approach with a fixed bin number *B*. If denoting the probability of each bin by $\{p_t,t=1,2,\ldots ,B\}$, DistEn is then calculated by the formula for Shannon entropy [13]:

5 $$\begin{aligned} \text {DistEn}(m,\tau ,B)=-\frac{1}{\log _2(B)}\sum _{t=1}^{B}p_t\log _2(p_t). \end{aligned}$$

### Parameters for entropy metrics

Common parameters shared by all these entropy metrics include the embedding dimension (or motif length) *m* and the time delay variable $\tau$. ApEn, SampEn, and FuzzyEn also share a threshold parameter *r*. A quantification level $\xi$ is needed in CE, and a bin number *B* is required in DistEn.

## Implementation

The main idea behind *EZ Entropy* software is to facilitate investigators not in engineering field, e.g., physiologists and clinicians, to perform such kind of analysis, so as to help promote the concept of nonlinear dynamical analysis in medicine field. In this section, I will introduce this software from several different aspects including system requirements, graphical user interface, functionality, setting, data import, results display, and results export.

### System requirements

*EZ Entropy* was developed using *App Designer* in MATLAB^®^ environment. However, with a standalone installation, the MATLAB^®^ environment is not necessary to use *EZ Entropy*. If a MATLAB^®^ based installation is preferred, MATLAB^®^ R2018a and later releases are recommended. Installing *EZ Entropy* based on older versions of MATLAB^®^ may cause unknown compilation problems since some new features of *App Designer* that are required in running this software are only available since early 2018. *EZ Entropy* will also be compiled as a web-based app and hosted in MATLAB^®^ Web App Server, such that users could easily run it in a web browser.

### The graphical user interface

**Fig. 1:**
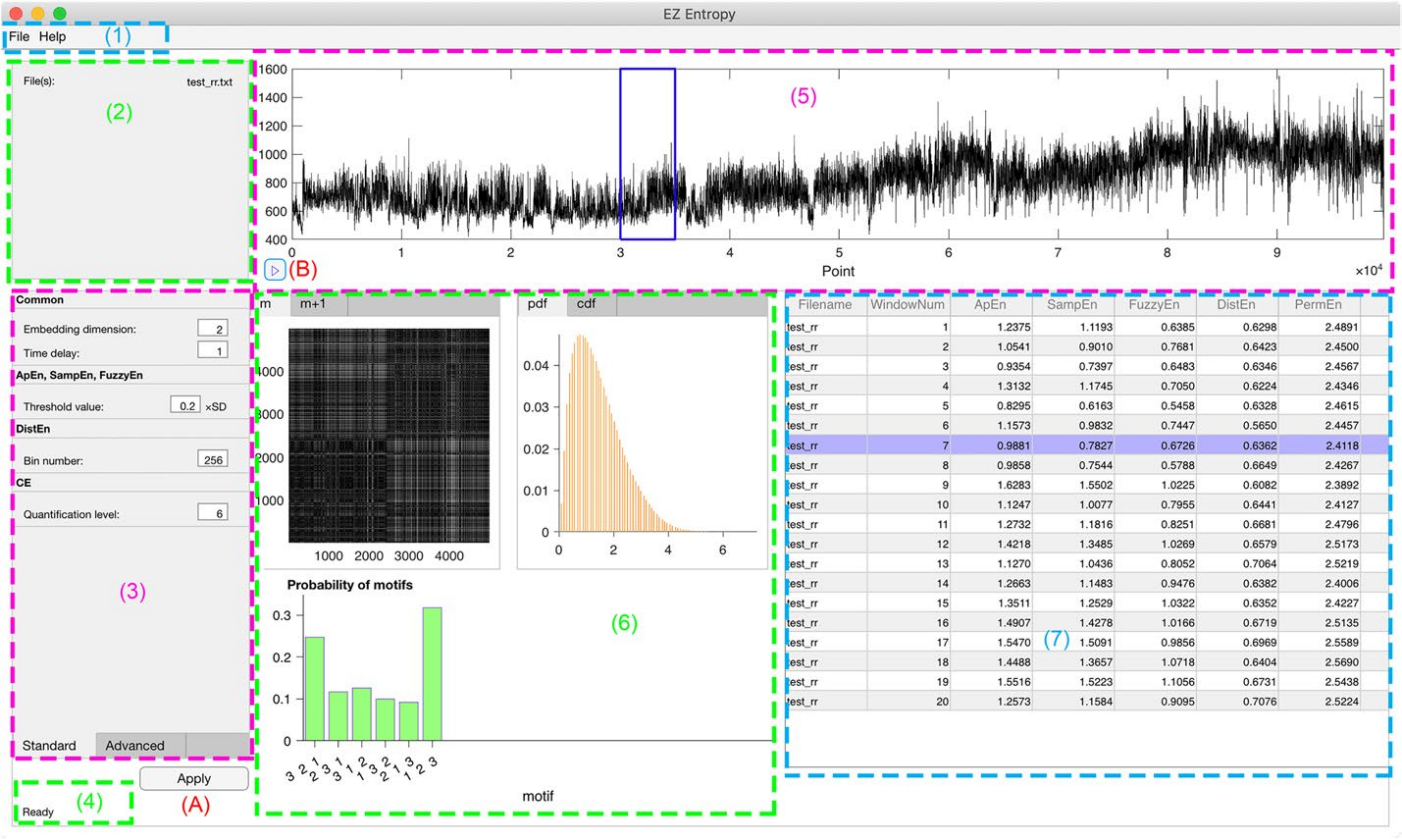
The graphical user interface of EZ Entropy. Regions: (1) menu; (2) file info; (3) setting; (4) status; (5) data display; (6) display of intermediate results; (7) results table. Buttons: (A) to execute; (B) to recall. Data and results are from an actual run

Figure 1 shows the main user interface through which users can interact with the software. There are mainly seven regions as illustrated in Fig. 1: (1) menu; (2) file info; (3) setting; (4) status; (5) data display; (6) display of intermediate results; and (7) results table. Regions (1) and (3) accept users inputs while regions (2), (4), (5–7) are for outputs. The button (A) is to execute the analysis after importing data and necessary settings. The button (B) is for recall purposes. Note that there are three tab controls [one in region (3) and two in region (6)] while in Fig. 1 only one tab for each is shown to give an overall view of the software interface. The other tab will be shown later in subsections below.

**Fig. 2:**
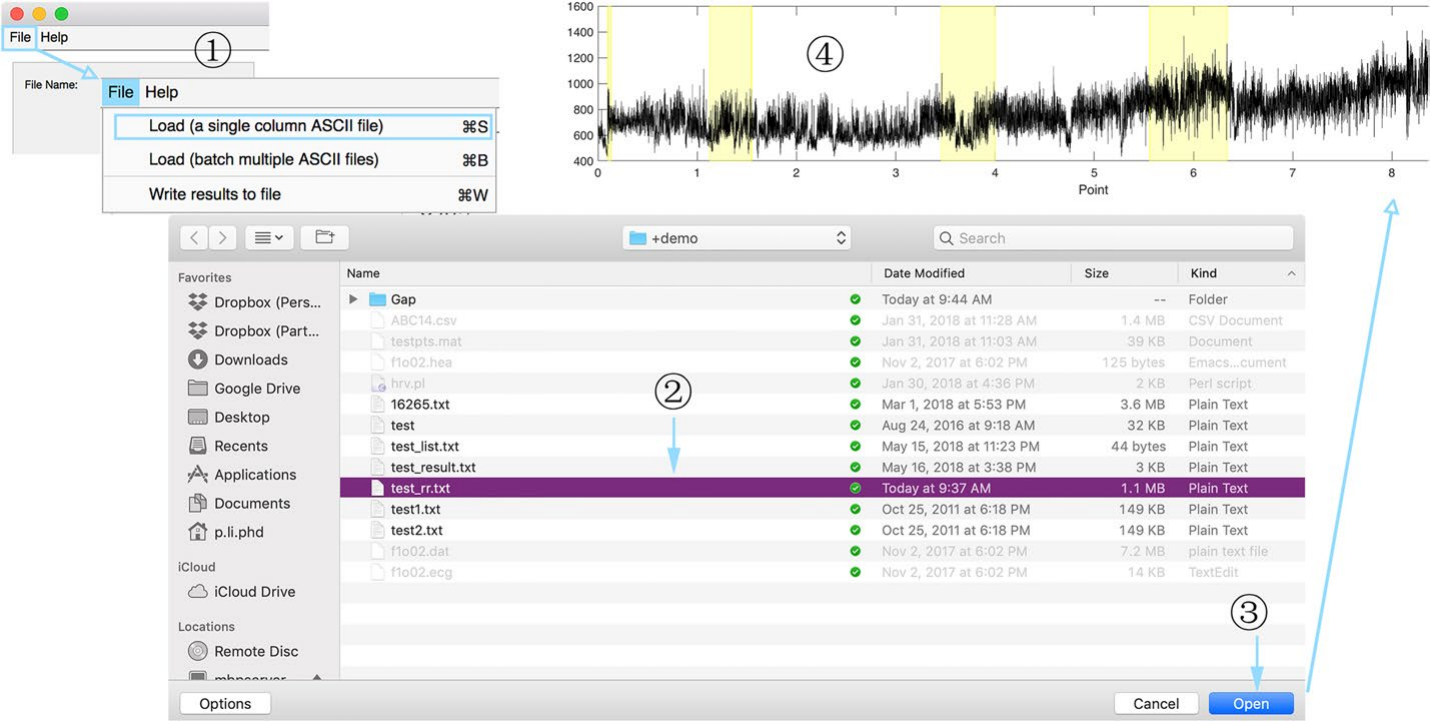
Steps for single recording analysis. Steps ①–③ demonstrate users’ operations while step ④ shows the signal imported

### Functionality

#### Single recording analysis

*EZ Entropy* can easily analyze a single signal recording (saved in a file of a certain format) and calculate the entropy results based on users’ setting with just several clicks. Figure 2 shows how to activate this function. After importing data, users need to define the input parameters and some running options through the *Setting* region. To perform the analysis on the data imported based on these parameters and options, users just need to simply click the button *Apply*. The software will do the analysis based on default parameters and options should users skip the settings and go directly click the *Apply* button.

#### Batch processing of multiple recordings

**Fig. 3:**
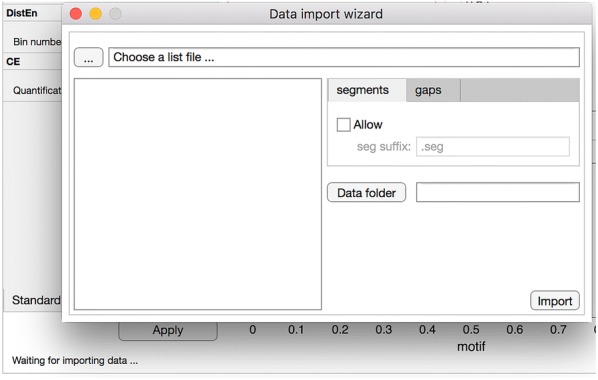
Data import wizard

Batch processing can be activated by clicking on the second item of “File” menu, as shown in Fig. 2. The *Data import wizard* will be popped out (Fig. 3). Click on button “...” and choose the list file which lists the names of all data recordings to be analyzed. These names of recordings will be shown in the list box on the left-hand side of data import wizard. The directory where these data recordings are located needs to be specified by clicking on the “Data folder” button. Two tab controls called “segments” and “gaps” are for two optional analysis configurations which will be detailed in “*Consideration of data quality*”. After all these, users click on button “Import” to close the wizard and migrate these configurations to the main interface.

### Data import

#### Data format

Only ASCII files with one column specifying the signal recording are accepted in the current release of *EZ Entropy*.

#### Import of single recording

As specified in “*Single recording analysis*”, a single ASCII file can be loaded by clicking on the menu item “Load (a single column ASCII file)” under “File” menu (see Fig. 2).

**Fig. 4:**
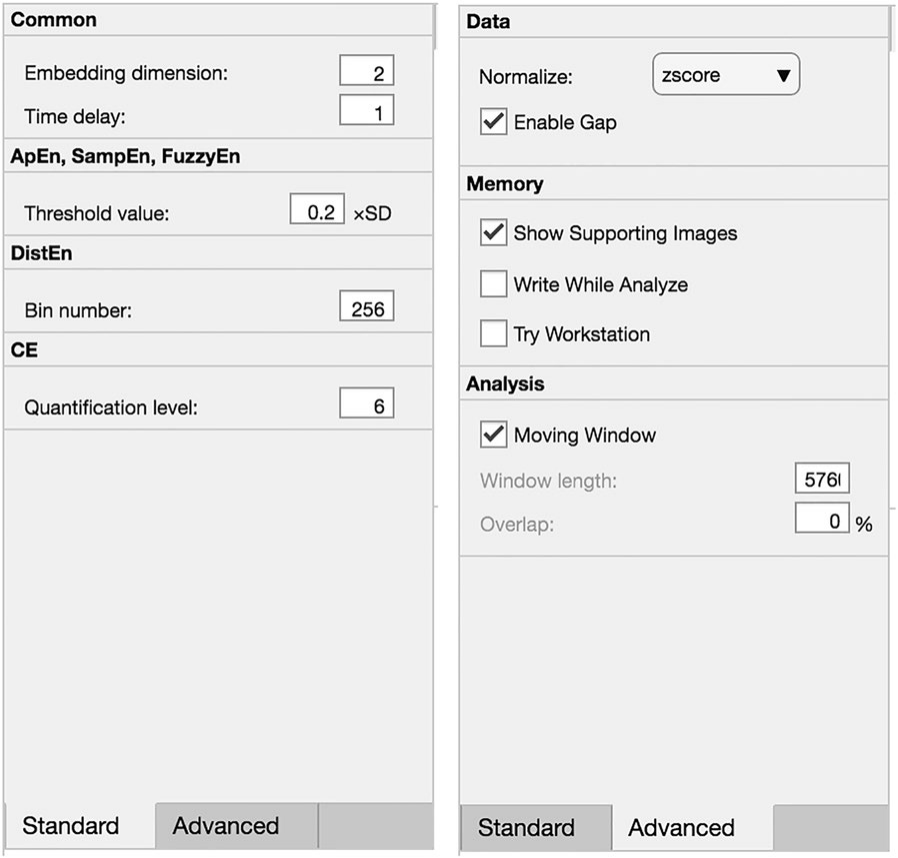
Shown the two tabs in region (3) of Fig. 1

#### Import of multiple recordings

Import of multiple recordings can be done with the help of the “Data import wizard” as specified in “*Batch processing of multiple recordings*” and Fig. 3.

### Setting

Figure 4 specifies the two tabs, namely “Standard” and “Advanced”, in region (3) of Fig. 1. Tab “Standard” is for defining parameters (for details, see “Parameters for entropy metrics”) and the “Advanced” tab is for further settings that will affect the calculations of entropy metrics.

**Fig. 5:**
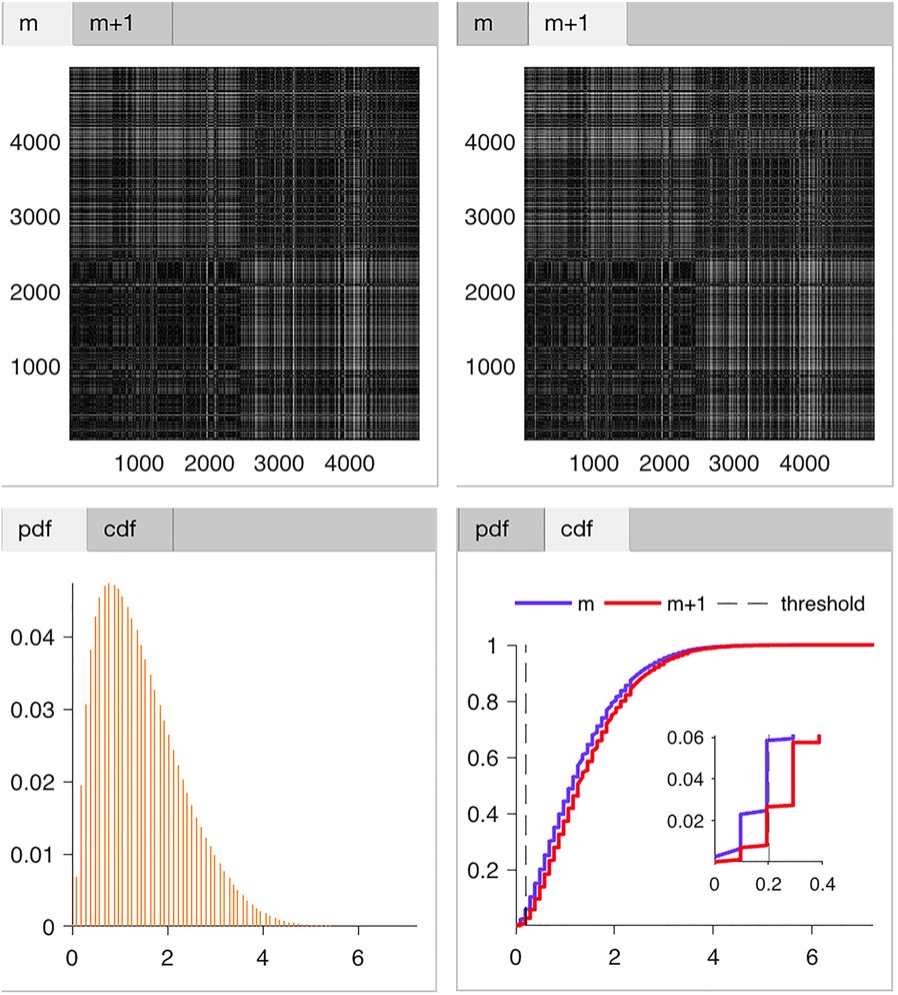
Display of intermediate results. Shown the distance map of embedding dimension *m* (upper left), $m+1$ (upper right), as well as the probability density function of embedding dimension *m* and $m+1$ in different colors (lower left), and the cumulative distribution function of dimension *m* and $m+1$ in different colors (lower right). For upper panels, both x- and y-axis are for the time point (or vector index). For lower panels, the x-axis is for the distance and y-axis for the probability

**Fig. 6:**
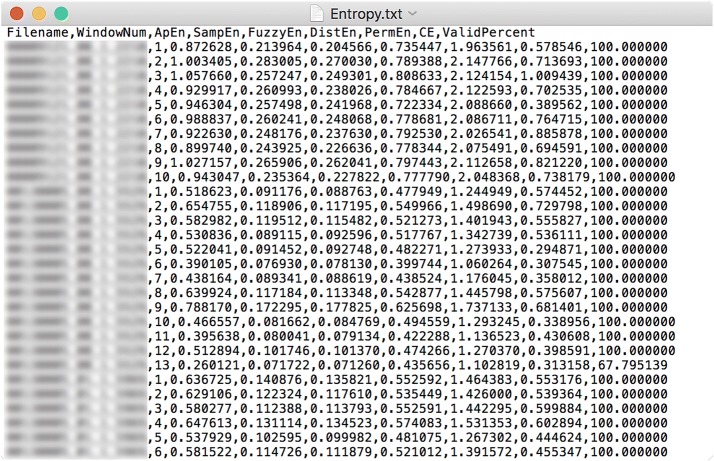
An example of the output file. The file names are delibrately blurred here

#### Consideration of data quality

This is the first item in the “Advanced” tab that can be further configured. The software accepts this input only in the context of single recording analysis. Users can select either “zscore” or “min–max” for data normalization. In addition, users could check or un-check the “Enable Gap” check-box. When the “Enable Gap” is checked, the software automatically searches within a sub-folder named “Gap” under the folder where the data recording is located whether there is a file named with the same name as the data recording while with an extension of “.gap”.

When users are batch processing multiple recordings, the consideration of data gaps are defined when importing data with the “Data import wizard”.

The file defining data gaps is also in ASCII format and is with two columns. The first column defines the starting points of data gaps while the second column specifies the end points of gaps. The number of rows is essentially equal to the number of gaps. The software automatically ignores this configuration if the specific gap file cannot be located. However, if the gap file is successfully loaded, the gaps will be highlighted in yellow background in the data display region [region (5) in Fig. 1; an example is shown in Fig. 2] and will be skipped (i.e., re-defined as not-a-number) when calculating the entropy metrics.

#### Consideration of running on a workstation or server

The software will stop calculating or seek further configuration about sliding windows when the data recording is too long (e.g., $>10,000$ points) as a contemporary self-protective way in case of memory leakage when using a personal computer. However, if users are using workstation or server, or are very confident about the resources of their hardware, they can check the “Try Workstation” check-box to force the software to do the calculation. On the other hand, if users worry too much about the possibility of memory leakage especially when batch processing multiple recordings, they can either un-check the “Show Supporting Images” check-box or check the “Write While Analyze” check-box. The first option will stop the software from displaying intermediate results using images, and the second option will avoid displaying too many lines in the results table by writing results to hard disk.

#### Sliding window analysis

The option of moving window is very helpful for the analysis of longer recordings. *EZ Entropy* offers the option of slide window analysis as a further setting option under the “Advanced” tab. By checking the “Moving Window” check-box, users could be able to define the window length (in points) and overlap (in percentage) between windows.

This option is acceptable for both single recording analysis and batching multiple recordings options. However, under the context of single recording analysis, an extra bonus offered by this software is that the check of “Moving Window” check-box will enable the recall button [button (B) in Fig. 1], meaning that users could replay the intermediate results for each window by clicking on this button. Figure 1 shows an example with recall button enabled. After click on the recall button, the corresponding window is boxed and the corresponding result line is highlighted in purple. All intermediate results corresponding to this window are displayed in region (6) of Fig. 1.

### Results display

#### Intermediate results

*EZ Entropy* uniquely offers the option to display intermediate results in image format including the distance map [13], the empirical probability density function (PDF) of distance [13], the cumulative distribution function (CDF) of distance [13], and the probability of signal motifs. The distance map, PDF, and CDF are available for both embedding dimensions $m$ and $m+1$, as shown in Fig. 5. These extra outputs may potentially be able to trigger new thoughts and ideas on the development or refinement of algorithms. For example, one idea that I am exploring right now is to treat the distance map as an image while using established artificial neural network algorithms (e.g., deep convolutional network) to characterize its patterns corresponding to healthy and diseased conditions.

#### Results table for entropy metrics

The calculation results will be displayed immediately in the UI-table control in region (7) of Fig. 1. By default the results for all files (when performing batch analysis) and all windows (when performing moving window analysis) are shown. However, if the “Write While Analyze” is checked (see “Consideration of running on a workstation or server” section), this results table will only display results for one file and will be refreshed and cleared after results being written to hard disk in order to show results of the next file.

### Exporting results

#### Exporting results manually

All results that are shown in the results table can be written to a file by clicking on the third menu item—“Write results to file”—of “File” menu, as shown in Fig. 2. The standard “Save As ...” dialog box will pop up and let users define the file name and directory. Results are saved in ASCII files with a header line specifying columns’ names and with multiple columns separated by comma, as shown in Fig. 6.

#### Exporting results automatically—export while analyze

This option can be enabled if the “Write While Analyze” check-box is checked. The standard “Save As ...” dialog box will pop up on checking of this check-box, so as to let users determine the file name and directory. Note this option should be set prior to performing the calculation (i.e., clicking on button “Apply”).

### Help

There are two items under the “Help” menu—“Documentation” and “About EZ Entropy”. The help document (the user manual in .pdf format) will be opened outside of this software using a PDF reader if installed with clicking on “Documentation”. The “About” dialog will be popped out after clicking “About EZ Entropy”.

## Results and discussion

### Sample run

Two examples runs, specifically, one for an example of analyzing a single data file and one for an example of batch processing multiple files, have been recorded and shown in Additional file 1: Movie S1. In each sample run, these previously mentioned settings will be touched, too.

### Availability of the software

Upon publication, request of *EZ Entropy* software can be addressed to me (e-mail to pli@sdu.edu.cn) and installation package will be sent free of charge for non-commercial use to researchers and clinicians, etc. The web-based *EZ Entropy* App will also be hosted in MATLAB Web App Server upon publication.

### Discussion

Entropy analysis of physiological time-series has been a hot topic in biomedical science and engineering fields, being capable of capturing unique, valuable, and additional characteristics hidden in the time-series that are not visually identifiable [5–17]. It usually requires at least some basic programming or coding knowledge to perform such kind of analysis as up to now no commercialized software has ever had these functions built in. This causes an obvious barrier to most clinicians and physiologists who are otherwise not trained in programming while on the other hand have plenty of data that may lead to new, useful observations should this novel analysis be performed.

To facilitate this, a software application—*EZ Entropy*—for the entropy analysis of physiological time-series, and certainly other physical time-series, was introduced. It is an easy-to-operate software application requiring only several clicks to perform the calculation. Besides, it offers different analysis options that are commonly applied when performing these calculations.

### Highlights

There are a couple of features, operation*-wise* and code*-wise*, that should be highlighted.

- Being built specifically in the context of entropy analysis and thus being extraordinarily focused
- Being highly interactive
- Offering options for both single file analysis and batch analysis
- Offering settings that are straightforward and easy to enable/disable
- Displaying intermediate results using image in order to trigger new methodological thoughts and ideas
- Exporting results in ASCII format that can be easily opened by almost all statistical software
- Displaying status message that has users notified in real-time about the progression
- Being programmed in complete object-oriented manner thus being quite easy to manage, maintain, and extend.

### Features to be added

This is the first release of *EZ Entropy* software with a few common options/settings. Yet there are plenty of other functions to be added to make this software more professional. Below I listed some features that will be enabled in future versions.

- Entropy analysis for multivariate data [35, 36]
- Multiscale analysis [37]
- Tolerance to other input data formats
- Ability of incorporating user-defined entropy metrics.

The ability to incorporate user-defined entropy algorithms as listed above should also be feasible even though it is only prospected at the time of this publication. Such a second development function is considered quite important, too, for even commercialized software. In the field of entropy analysis, there are many novel algorithms being developed by biomedical engineers [14, 18–24]. This software application with such a second development capability will significantly help promote the application of new entropy algorithms in physiology and clinical medicine.

In addition, error handling capability is also quite important to render software stability and compatibility. *EZ Entropy* takes full advantage of MATLAB’s error handling logic such that error or warning messages will be prompted in the MATLAB command window should there be any invalid inputs. The messages are quite straightforward for even non-technicians to follow. They will show details about why an error occurs and where it is. However, it is worth noting that this is actually rare since all input parameters have predefined default values and other calculation settings are by clicking instead of typing in which reduces the possibility of accidentally introducing invalid configurations. To improve the completeness, the function of the “Status” bar (see Fig. 1) will be expanded in future to also show prompts of possible invalid operations.

It is worth noting that MATLAB provide a “.fig” file in order to export both the user interface and data together to local hard disk. This is kind of equivalent as saving the workspace as a project that is able to be worked on again next time when opening it. It is possible to add an additional menu item to achieve this. However, it might make more sense to save the workspace when batch processing multiple data files. For example, the users may want to occasionally interrupt the work-flow and continue later. To achieve this, the software needs to be able to listen to some event (i.e., interrupt) and takes appropriate actions to handle the event. This is unfortunately not supported by MATLAB since it is not a multithreaded programming language. It cannot handle any new event until the current one (e.g., a loop) is finished.

## Conclusions

With the rapid developments in new data mining theories and technologies, there might be many new properties hidden in the seemingly simple physiological signals to be detected. These new properties may emerge as valuable features for the evaluation of health status or may complement previously identified features. Either way, researchers in the related fields should be encouraged to be always open-minded to the fresh young blood. Specialized software, for example, the heart rate variability analysis software previously published [38] and the *EZ Entropy* software introduced here, will certainly fill the needs and potentially play an increasingly important role in the scientific community.

### Availability and requirements

Project name: *EZ Entropy* software implementation
Project home page: N/A
Operating system(s): Platform independent
Programming language: MATLAB
Other requirements: requires MATLAB R2018a and later releases
License: GNU GPL
Any restrictions to use by non-academics: licence needed.

## Additional file

**Additional file 1: Movie S1.** Demonstration and sample runs of using *EZ Entropy*.
